# miR-155 aberrant expression impairs tumor rejection because of its targeting of ICOSL and multiple pathways implicated in the antitumor response

**DOI:** 10.1016/j.pharmr.2025.100088

**Published:** 2025-03-09

**Authors:** Esmerina Tili, Jean-Jacques Michaille, Carlo M. Croce

**Affiliations:** 1 Department of Anesthesiology, Wexner Medical Center, College of Medicine, The Ohio State University, Columbus, Ohio; 2 The Ohio State University Comprehensive Cancer Center, Columbus, Ohio; 3 Department of Cancer Biology and Genetics, College of Medicine, The Ohio State University, Columbus, Ohio

## Abstract

Cancer treatments have dramatically improved because of advances in surgery, radiotherapy, and chemotherapy. Although the duration of remission has steadily increased in recent years, preventing metastasis and achieving complete remission is still beyond reach for various types of cancers. However, recent advancements in immunology have facilitated the development of immunotherapies aimed at enhancing the specificity and efficacy of natural anticancer immune responses while impairing the inhibitory effects of immune checkpoints. Although immunotherapies combined with other treatment modalities have already produced remarkable results in previously untreatable tumors, many patients still do not achieve complete remission. In this review, we explore the effects of miR-155, a microRNA that plays a critical role in initiation and resolution of both innate and adaptive immunity. Among the many target transcripts of miR-155 are those encoding immune checkpoints, cell cycle regulators, epigenetics regulators, transcription factors, DNA repairs factors, and factors involved in various signaling pathways. The inhibitory effects of miR-155 on its target transcripts are likely to be context- and dose-dependent. As certain miR-155 targets can have opposing effects based on their dose and activity, therapies aimed at increasing or decreasing miR-155 levels can potentially backfire, inhibiting the beneficial effects of widely used anticancer drugs. Precise monitoring and adjustment of miR-155 levels, depending on the type and stage of tumors, should enhance the effectiveness of immunotherapies and increase the percentage of patients achieving complete remission in the future, particularly when immunotherapies are combined with chemotherapies.

**Significance Statement:**

Although immunotherapies developed the last decade have brought hope and improved cancer treatments, prevented metastasis, and increased the rate of complete remission, many aspects of the anticancer immune response are controlled by miR-155, a microRNA whose activity is both context- and dose-dependent. Therefore, it is essential to determine the optimal levels of miR-155 activity according to the type and stage of tumors, in order to fully unlock the potential of immunotherapies in combination with surgery, radiotherapies, or chemotherapies.

## I Advances in cancer immunotherapy

Immunotherapies have revolutionized cancer treatment; however, only a minority of patients achieve successful responses, indicating an urgent need for further optimization. For instance, only 22% of patients demonstrated durable responses lasting over 10 years after receiving anti–cytotoxic T-lymphocyte antigen 4 (CTLA-4/CD152), the first immune checkpoint inhibitor (ICI) tested against melanoma.^1^, ^2^, ^3^, ^4^ Similarly, although anti–programmed death 1 (PD-1; PDCD1; CD279) and anti-programmed death ligand 1 (PD-L1; CD274) inhibitors have shown efficiency in numerous patients, the reasons behind the lack of effective responses in some individuals remain incompletely understood.^5^^,^^6^ In a recent phase II clinical trial for solid tumors (investigator-initiated phase 2 study of pembrolizumab immunological response evaluation, NCT02644369) evaluating the PD-1 inhibitor pembrolizumab in immunotherapy-naïve patients with advanced or metastatic solid tumors including head and neck squamous cell carcinoma, high-grade serous ovarian carcinoma, triple-negative breast cancer (TNBC), and metastatic melanoma, responses were observed only in 24% of the patients with tumors categorized as “hot”—ie, tumor expected to induce a strong immune response—yielding a median overall survival (OS) of 20.4 months. In contrast, only 10% of patients with “cold” tumors responded, resulting in a poor OS of 7.14 months.^7^

Numerous factors influence the responses to ICIs and overall patient outcomes: (1) tumor characteristics, including the genetic landscape of tumor cells, tumor antigens, antigen-presenting major histocompatibility complex (MHC) proteins, and receptor/ligand interactions in tumor cells; (2) the host’s immune background, encompassing the presence of various T-cell subtypes, particularly levels of CD8+ tumor-infiltrating lymphocytes, and the involvement of other immune cells (both local and infiltrating) within the tumor microenvironment (TME), such as the myeloid-derived suppressor cells (MDSCs); and (3) the overall strength of the immune response to tumor cells. Consequently, tumor cells can employ a variety of immune evasion strategies that allow them to evade treatment. For example, the deletion of PD-L1 gene in certain head and neck cancers likely explains the lack of immune response to PD-L1 inhibitors,**^8^** rendering anti-PD-L1 treatment irrelevant if PD-L1 is deleted or not expressed. Moreover, as most patients undergo combined radiotherapy and chemotherapy, understanding the impact of radiotherapy on tumor antigens, their presentation and recognition, as well as the abscopal effects when combined with immunotherapy, is critical for achieving therapeutic success. Similarly, the downregulation of inducible T-cell costimulator ligand (ICOSL/ICOSLG/CD275) expression can hinder its binding to inducible T-cell costimulator (ICOS/CD278), a crucial factor for T-cell activation. This disruption of ICOSL binding to ICOS subsequently impairs T-cell activation and their binding to tumor cells, thereby compromising the cytotoxic response and facilitating tumor cell evasion. Therefore, the individual assessment of patients’ genetic landscape, coupled with the immune phenotype of tumor cells and characterization of tumor-infiltrating lymphocytes and TME, has become essential for predicting and improving responses to immunotherapies. Overall, further research is needed to enhance and reinforce antitumor immune responses in order to improve the outcomes for all patients with cancer.

The advent of immunotherapies began with the identification of key ligands and receptors that function as either activators or inhibitors, playing crucial roles in modulating T-cell responses to multiple challenges. Current immunotherapeutic approaches include monoclonal antibodies against ICIs, chimeric antigen receptor T-cell therapy, cancer vaccines, oncolytic virus therapy, cytokine therapy, adoptive cell transfer, and the use of immune modulators, all designed to enhance the body’s capacity to recognize and target cancer cells. In this review, we will focus on ICIs and how the microRNA miR-155 may serve as a determinant for their success or failure through its multifaceted effects within tumor cells (intrinsic effects) and its regulation of the immune response against tumor cells (extrinsic effects).

## II miR-155 and cancer

Abnormal expression of microRNAs is observed in various pathologies, particularly in cancer. miR-155 plays a crucial role in modulating both innate and adaptive immune responses, with elevated levels of miR-155 found in numerous hematological malignancies and solid tumors. Studies have identified multiple targets of this microRNA, primarily elucidating its intrinsic effects within cancer cells, such as the regulation of cell proliferation, differentiation, and survival. Our recent research has focused on how the dysregulation of miR-155 expression in different malignancies affects the host’s immune response against cancer cells, with the aim of uncovering novel immune-related therapeutic targets.^9^ Specifically, we have demonstrated that miR-155 targets and reduces the expression of ICOSL,^9^ which along with ICOS forms a costimulatory immune checkpoint. In 2 different mouse models of miR-155 overexpression in B cells, we found that lymphoma B cells expressing ICOSL are eliminated, whereas those that are ICOSL-negative continue to survive and proliferate. This finding suggests that miR-155 expression in tumor cells may impair tumor rejection by targeting critical factors involved in T-cell activation and T–cell–dependent killing of cancer cells.

**Fig. 1:**
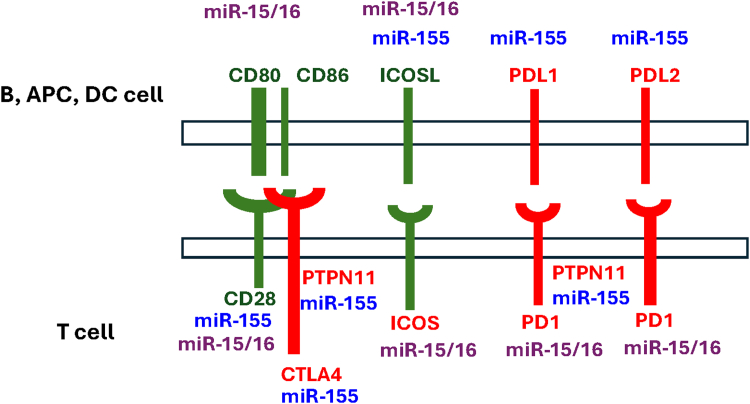
Interplay of miR-155 and miR-15/16 in immune checkpoints. miR-155 and miR-15/16 have been shown to or are predicted (based on Targetscan software^10^); to target, transcripts encoding factors that are critical for T-cell activation (marked in green) and inhibition (marked in red). This suggests that these microRNAs play a key role in fine-tuning the activity of immune checkpoints. The dysregulation of these microRNAs in cancer has important implications for antitumor immunity and tumor progression. Notably, the expression of miR-155 is frequently elevated in various malignancies, whereas miR-15/16 are often either deleted or downregulated. Additionally, the binding sites for miR-15/16 in CD80 and PDCD1 transcripts are exclusively found in mammals, conferring a great evolutionary significance to the accumulation of regulatory effects by these microRNAs in T-cell regulation within this taxon. PTPN11/SHP2-phosphatase; PDL2/CD273/PDCD1LG2; CD80/B7-1; CD86/B7-2; ICOSL/B7-H2/CD275; PDL1/B7-H1/CD274. APC, antigen-presenting cells; DC, dendritic cells.

In addition to miR-155, other microRNAs are anticipated to modulate the expression of immune checkpoints (Fig. 1).^10^ Notably, the miR-15/16 cluster is frequently deleted in various malignancies, including chronic lymphocytic leukemia (CLL),^11^ impairing the targeting of PD-L1, and resulting in elevated levels of this inhibitory checkpoint in tumor cells that harbor the deletion of these microRNAs.^12^ Consequently, a comprehensive understanding of the molecular mechanisms linking the dysregulation of miR-155 and other microRNAs in cancer with immune checkpoints has the potential to uncover novel therapeutic targets for patients who do not respond adequately to existing immunotherapies. Such insights may elucidate the mechanisms underlying deficient tumor rejection and resistance, as well as the limited responses to immunotherapies. Moreover, the levels of expression of these microRNAs within tumors may be critical for predicting and assessing the effectiveness of ICIs. Although the involvement of the ICOS/ICOSL signaling pathway in immune surveillance and deficiency has been well documented, this is the first review to explore the functional links between the ICOS/ICOSL signaling pathway and miR-155.

## III miR-155 as a master regulator of the immune response

miR-155 began to garner significant attention from biologists after our group, along with Dr Baltimore’s team, established its roles in inflammation and innate immune responses downstream of lipopolysaccharides (LPS), polyriboinosinic:polyribocytidylic acid, and interferon (IFN) signaling in macrophages and monocytes.^13^^,^^14^ Subsequently, both groups reported that miR-155 targets SHIP1/INPP5D (inositol polyphosphate-5-phosphatase D), a phosphatase that negatively regulates the phosphoinositide 3-kinase (PI3K) pathway in B cells and other immune cells. This interaction endows miR-155 with a potent proliferative activity during the immune response.^15^^,^^16^ Since then, several additional transcripts that are targets of miR-155 have been identified, including those encoding CCAAT/enhancer binding protein *β* (C/EBP*β*), a lineage-specific transcription factor involved in the myeloid development pathway,^15^ purine-rich box 1 transcription factor critical for B-cell maturation,^17^^,^^18^ as well as QKI (KH domain containing RNA binding protein), which is implicated in RNA processing.^19^ A comprehensive list of other targets can be found in the reviews of Tili et al^20^^,^^21^ and Michaille et al.^22^

Building on our discovery that miR-155 levels are elevated in CLL,^23^ we developed a transgenic mouse model —the first engineered mouse model for a microRNA—in which the expression of a miR-155 transgene is regulated by the variable region of immunoglobulin heavy chain region of E*μ* enhancer. This construct drives high levels of miR-155 expression specifically in B cells.^24^ This mouse has enabled us to investigate the consequences of elevated levels of miR-155 in B-cell leukemias and lymphomas. Our model mice specifically replicate B-cell malignancies characterized by high levels of miR-155. Notably, mice overexpressing miR-155 in B cells show an early phenotype of preleukemic phenotype, characterized by pre-B-cell proliferation in the bone marrow and spleen, which subsequently progresses to overt B-cell malignancy.^24^ In these mice, the early preleukemic phenotype may arise from the intrinsic effects of miR-155, while the development of frank B-cell malignancy likely reflects the immune system’s failure to recognize and eliminate malignant cells that have downregulated ICOSL expression.^9^ Following our work, other groups developed transgenic or knockout mouse models to study the role of miR-155 in other malignancies, as well as in neurodegenerative and cardiovascular pathologies. Of particular interest is an elegant mouse model of miR-155 controlled expression developed by Dr. Slack’s group, which allows for inducible and tissue-specific overexpression of miR-155.^25^

miR-155 is also expressed in natural killer (NK)–cell lymphoma-derived cell lines.^26^ Its overexpression under the Lck promoter to drive the expression of this microRNA in mouse T-cell lineage led to NK cell expansion, arrest in NK cell terminal differentiation, and their functional activation.^27^ Two specific targets of miR-155 identified in invariant NKT cells are ETS1 and ITK transcripts, which were previously reported to encode factors implicated in invariant NKT cell differentiation.^28^ Additionally, miR-155 is expressed at elevated levels in regulatory T cells (Tregs) under the transcriptional control of forkhead box P3 (FOXP3).^29^ In various cell types, including T cells, miR-155 targets *SOCS1* transcripts, thereby enhancing the proliferation of Tregs during immune response.^29^ The reduced expression of CTLA-4 in Tregs, as a consequence of being targeted by miR-155, has been associated with poor prognosis in patients with metastatic melanoma.^30^ Conversely, in a mouse B16 melanoma model, the expression levels of miR-155 in CD8+ T cells were found to be dependent on antigen affinity and dose, with higher levels of miR-155 correlating with increased cytokine production and improved tumor control.^31^ Accordingly, in melanoma patients, a positive correlation was observed between the levels of miR-155 in human effector memory CD8+ T cells and the frequency of these cells in tumor-infiltrated lymph nodes.^31^ Furthermore, to enhance IFN*γ* production and restrain syngeneic tumor growth, T cells within the TME must express miR-155.^32^ Consistent with the finding that miR-155 targets CTLA-4 transcripts,^30^ the beneficial effects of miR-155 expression in T cells are underscored by observations in miR-155 T-cell-conditional knockout mice. In these mice, the administration of ICIs targeting CTLA-4 or PD-1/PD-L1 was needed to increase the number of T cells expressing IFN*γ*, activate tumor-associated macrophages, and enhance the expression of effector genes.^32^

Finally, the transcription factor C/EBP*β* plays a significant role in immune and inflammatory responses exhibiting pro- or antioncogenic activities depending on the cell type and the isoform expressed (reviewed by Tolomeo and Grimaudo^33^). C/EBP*β* is targeted by miR-155 not only in B cells^15^ but also in MDSCs.**^34^** Elevated levels of miR-155 have been observed in the myeloid cells of mice with conditional deletion of the *SOCS3* gene, which permits miR-155 to target *C/EBPβ* transcripts. This targeting leads to enhanced activation of the Wnt/mTOR signaling pathway, resulting in the development of MDSCs with reduced autophagic capabilities. Consequently, this allows murine EO771 breast cancer cells injected into the mammary fat pad to form tumors.^34^

Overall, miR-155 expression in T cells, and other immune cells present within the TME, such as B and myeloid cells, can potentially help immune cells to better recognize and eliminate tumor cells. These effects are likely attributable to the positive effects of miR-155 on cytokine production, including tumor necrosis factor (TNF),^13^ IFN*γ*,^35^ and other cytokines regulated by suppressors of cytokine signaling (SOCSs) factors. Nevertheless, the mechanistic bases of miR-155’s positive effects in immune cells within the TME remain poorly understood, particularly in the context of responses to ICIs treatments, which have become the standard of care for several cancers.^36^

## IV Intrinsic effects of miR-155

In our pioneering publication profiling the expression levels of then-known microRNAs across 6 solid tumors, we found that miR-155 expression was significantly elevated in breast, colon, and lung cancers.^37^ Subsequent research revealed that miR-155 levels are also increased in other solid tumors, including pancreatic, cervical, and thyroid cancers.^38^, ^39^, ^40^ High levels of miR-155 have also been reported in hematological malignancies such as CLL,^23^^,^^41^ diffuse large B-cell lymphoma (DLBCL),^42^, ^43^, ^44^ follicular center lymphoma,^43^ Hodgkin lymphoma,^45^ and acute myeloid leukemia.^46^^,^^47^ Unfortunately, elevated expression of miR-155 in CLL and other malignancies is often associated with poor prognosis,^23^^,^^48^^,^^49^ suggesting a deleterious role for this microRNA in these pathologies. Accordingly, high levels of miR-155 have been shown to favor chemoresistance in lung, breast, colon, and other cancers (reviewed by Bayraktar and Roosbroeck^50^). In lung cancer, miR-155 targets transcripts encoding the transcription factor FOXO3a, whose expression, controlled by NF-*κ*B activity,^51^ positively correlates with sensitivity to tyrosine kinase inhibitors and improved progression-free survival in patients, independent of epidermal growth factor receptor mutations. Consequently, the downregulation of FOXO3a expression because of miR-155 activity increases the resistance of lung cancer cell lines to the epidermal growth factor tyrosine kinase inhibitor gefitinib.^51^ Furthermore, miR-155 overexpression may promote genomic instability by repressing FOXO3a and, consequently, the expression of high-fidelity delta polymerase (POLD1), altogether leading to the activation of error-prone DNA double-strand break repair mechanisms.^52^ Targeting of FOXO3 by miR-155 has also been linked to the radioresistance of colorectal cancer (CRC) cells,^53^ and nasopharyngeal carcinoma (NPC) tissues, where miR-155 targets *Ubiquilin 1* transcripts, resulting in the activation of the PI3K-AKT pathway.^54^ Additionally, miR-155 reduces the levels of the kinase WEE1,^55^ which normally inhibits cell cycle progression at the G_2_/M phase by catalyzing the inhibitory tyrosine phosphorylation of the Cdc2/Cyclin B complex, depending on DNA integrity. This action induces a mutator phenotype in cells with high levels of miR-155.^55^ On the good side, the accumulation of unrepaired DNA in tumor cells can lead to the production of neoantigens, thereby increasing the immunogenicity of cancer cells.^56^ Moreover, the activation of the cGMP-AMP synthase/STING pathway by the presence of double-strand and single-strand DNA in the cytoplasm induces the IFN*γ* pathway, resulting in heightened immunogenicity of tumor cells.^57^, ^58^, ^59^ In addition to enhancing tumor immunogenicity, the targeting of RAD51 transcripts by miR-155 increases the radiosensitivity of TNBC cells.^60^ Reduced RAD51 activity inhibits homologous recombination, rendering these cells incapable of repairing the DNA damage caused by radiotherapy.^60^ Beyond RAD51, miR-155 also targets and downregulates the expression of other transcripts involved in DNA repair, including those encoding the mismatch repair factors hMSH2, hMSH6, and hMLH1.^61^ The seemingly beneficial and detrimental effects of miR-155 in cancer may be linked to the chronic levels it reaches in various malignancies, as discussed in our review by Tili et al^20^ and further examined in laboratory experiments conducted by Narayan et al.^62^

In transgenic mice with tetracycline-controlled expression of miR-155 (miR-155-Tet ON/OFF mice)^25^ overexpression of miR-155 resulted in a significant reduction of Ship1^25^ and Socs1^9^ expression in tumor cells. Turning ON miR-155 also led to the accumulation of miR-155-overexpressing tumor cells harboring mutations in the cellular-Kit proto-oncogene.^63^ In breast cancer, elevated levels of miR-155 positively correlated with enhanced antitumor immunity.^64^ When EO771 breast cancer cells overexpressing Bic—the host gene from which mature miR-155 is derived^42^—were injected into the mammary fat pad of mice, the increased levels of miR-155 delayed tumor progression, enhanced T-cell infiltration, and increased tumor sensitivity to ICIs.^64^ Additionally, EO771-Bic tumors exhibited a higher number of cancer cells undergoing apoptosis. Conversely, when EO771 cells were injected into global miR-155 knockout mice, the absence of miR-155 activity accelerated tumor growth.^64^ These findings suggest that miR-155 expression by breast cancer cells may suppress tumor growth by promoting immune cell infiltration. However, the increase in T-cell infiltration might not be sufficient, as the infiltrating T cells must recognize and bind to the tumor cells, in order to eliminate these cells.

## V miR-155 extrinsic effects on the anticancer immune response

The functions of miR-155 are shaped by various factors, including the stage of the tumor, the type of treatment a patient receives, the specific cell type in which it is expressed, and the levels of miR-155 achieved during cellular transformation. The functional outcomes are also contingent on the significance of the targeted genes related to cell survival and proliferation (intrinsic effects) as well as to cellular immunogenicity (extrinsic effects), which in turn impacts tumor cells recognition and elimination by cytotoxic T cells. For instance, mutations in tumor cells induced by miR-155’s targeting of DNA repair genes may enhance immunogenicity, making these cells more susceptible to immune attack. Conversely, miR-155 also targets ICOSL transcripts, impairing T cells’ ability to effectively recognize and destroy tumor cells, despite their newly acquired mutations. In this aspect, a significant gap in our understanding remains regarding the molecular mechanisms by which miR-155 modulates the antitumor immune response and tumor immunogenicity. Based on our recent data in mice, overexpressing ICOSL in tumor cells is anticipated to counteract the negative effects of miR-155 on tumor cell survival and proliferation, thereby rendering tumor cells more susceptible to elimination by T cells. Although the targeting of different DNA repair genes by miR-155 may enhance tumor cell immunogenicity, our experiments nevertheless showed that turning OFF the expression of miR-155 after it had been ON resulted in the rapid elimination of tumor cells in part due to the re-expression of ICOSL and reactivation of the ICOS/ICOSL immune checkpoint. These effects were further augmented by tumor cells undergoing apoptosis upon re-expression of Ship1^25^ or Socs1^9^ when miR-155 was turned OFF. Thus, the targeting of ICOSL by miR-155 is detrimental as it diminishes the capacity of the immune system to recognize and eliminate tumor cells characterized by high levels of miR-155. Additionally, miR-155 targeting of ICOSL can impair the recognition of potentially immunogenic tumor cells resulting from mutations accumulated because of miR-155 impact on DNA-repair genes. This scenario may lead to the accumulation of frustrated T cells that, despite activation, cannot effectively bind to and eliminate tumor cells, potentially resulting in T-cell exhaustion. Importantly, these findings do not imply that complete inhibition of miR-155 would be the optimal strategy. Instead, it may be desirable to maintain the levels of miR-155 within a yet-to-be-determined range that allows for sufficient inhibition of the DNA repair system to produce mutations that increase cell immunogenicity, while not impairing the activation of T-cell response by the ICOS/ICOSL pathway. This strategy will also be complemented with therapies delivering ICOSL to tumor cells that would promote their recognition and elimination by T cells. Notably, the expression of miR-155 is elevated in breast cancers associated with BRCA1 mutations. Since BRCA1 epigenetically inhibits the expression of miR-155,^65^ it is expected that the levels of ICOSL and consequently of T-cell activation would be reduced in these tumors. Therefore, breast cancer cells harboring BRCA1 mutations may respond favorably to immunotherapies, particularly those aimed at enhancing ICOSL expression. In another study, miR-155 was identified as one of several microRNAs present in melanoma-derived extracellular vesicles, which are responsible for converting monocytes into MDSCs.^66^ The levels of microRNAs, including miR-155, in these extracellular vesicles were found to be higher in circulating CD14-positive monocytes, as well as in plasma and tumor samples from melanoma patients.

## VI CD28, CTLA-4, and PD-1/PD-L1 immune checkpoints

Beyond ICOS, T-cell responses to tumor antigens or pathogens are regulated by several immune checkpoints that are critical for mounting an efficient immune response. This response protects the host from infectious agents and tumor cells while maintaining self-tolerance. Consequently, any malfunction in the immune checkpoint system can diminish the intensity and/or duration of the immune response, allowing infectious agents or cancer cells to overcome the body’s defenses. Conversely, it may also increase the intensity and/or duration of the immune response, potentially leading to tissue damage. It is well established that prolonged and exacerbated inflammation can have multiple deleterious consequences on organs such as heart, brain, liver, pancreas, and kidneys. Chronic inflammation can also exacerbate existent tumors or promote the formation of new tumors, particularly because of miR-155 targeting transcripts that code for tumor suppressor genes (as reviewed by Tili et al^20^^,^^22^ and Michaille et al^21^). Therefore, studying unique and nonredundant immune checkpoints,^67^ alongside the effects of dysregulated microRNAs in corresponding cancers that cause immune checkpoint malfunction, is essential for improving the immune checkpoint-based therapeutics for various malignancies.

Costimulation of T cells occurs primarily through the CD28 signal transduction pathway, whereas other cell surface receptors, including the CD28 homolog CTLA-4, also provide costimulatory signals. Both CD28 and CTLA-4 are glycoproteins expressed on the surface of T cells; however, the engagement of CD28 after antigen receptor activation delivers the critical costimulatory signal necessary for T-cell activation.^68^^,^^69^ CTLA-4 expression is the first to be upregulated after T-cell receptor (TCR) ligation.^70^^,^^71^ In the presence of optimal costimulation and Fc cross-linking (immunoglobulin fragment crystallizable), anti–CTLA-4 antibodies can inhibit T-cell proliferation, leading to downregulation of T-cell immune function.^72^ This early research led to the discovery that, once expressed at elevated levels, CTLA-4 competes with CD28 for binding to B7 (CD80/CD86), thereby reducing CD28-mediated costimulation of T cells in conjunction with MHC-I-presented antigens binding to the TCR.^70^^,^^72^^,^^73^ In contrast to CTLA-4, which is induced early following TCR activation, the expression of PD-1 arises at later stages and is associated with the recruitment of tyrosine phosphatases such as SHP-2,^74^, ^75^, ^76^ which attenuate downstream TCR activation and signaling.^77^ This biphasic induction—characterized by early CTLA-4 expression followed by late PD-1 expression—serves to prevent excessive and prolonged T-cell activation, while facilitating a return to a resting phase under normal conditions. The cellular sources of ligands that bind to CTLA-4 (CD80/CD86) and PD-1 (PD-L1) are distinct.^78^ In studies comparing MC38-derived CRC cells which are considered immunogenic to B16BL6 melanoma tumor cells which originate “cold” tumors when injected into syngeneic mice found that anti-CTLA-4 and anti-PD-1 therapies operate through distinct cellular mechanisms.^78^ Notably, anti-CTLA-4 but not anti-PD-1 impacted the CD4 effector compartment, by expanding an ICOS^+^ Th1-like CD4 effector subset, whereas anti–PD-1 predominantly induced the expansion of specific tumor-infiltrating exhausted-like CD8 T-cell subsets.

## VII ICOS/ICOSL immune checkpoint in cancer immune responses

ICOS and its ligand ICOSL represent an important immune checkpoint in cancer immune responses. ICOSL is a T-cell-specific surface costimulatory molecule structurally related to CD28 and CTLA-4.^79^ It plays a crucial role in regulating T-cell proliferation and cytokine production.^80^, ^81^, ^82^ Costimulation through ICOSL is essential for both T helper and B-cell functions during T-cell-dependent B-cell responses.^83^ ICOS is not expressed in resting cells but is induced following T-cell activation.^79^^,^^80^ The expression of ICOS by activated T cells is required for mounting effective T-cell-dependent immune responses.^84^ Notably, ICOS is highly expressed by T cells in germinal centers (GCs), where it is involved in T-B cell communications and T-cell-dependent B-cell responses.^85^ In healthy mice, ICOSL is expressed at basal levels in different lymphoid tissues and is particularly found on B lymphocytes, dendritic cells, macrophages, vascular endothelial cells, and epithelial cells.^80^^,^^86^, ^87^, ^88^, ^89^, ^90^, ^91^ Mice lacking either *Icos* or *IcosL* exhibit similar immune deficiencies primarily affecting T-cell-dependent B-cell responses. Specifically, ICOS-ICOSL signaling is critical for thymus-dependent antibody responses and for GC reaction, thus playing a critical role in T-cell development, activation, and function.^92^, ^93^, ^94^, ^95^, ^96^, ^97^, ^98^, ^99^ Interestingly, the generation of tissue-resident memory T cells is defective in *ICOS*^*−/−*^ CD8^+^ T cells; however, these cells are still capable of producing recirculating memory populations normally.^100^

Supporting the crucial role of ICOSL in the immune response, mutations in ICOSL gene have been identified in a male patient and an unrelated female patient. Specifically, a c.657C > G (p.N219K) mutation was found to abrogate cell surface expression of ICOSL, resulting in combined immunodeficiency.^101^^,^^102^ Both patients exhibited similar phenotypes associated with their combined immune deficiency syndrome. The first patient, a male presented with humoral immunodeficiency characterized by recurrent respiratory tract infections, compromised cell-mediated immunity causing extensive mucocutaneous human papillomavirus disease, and progressive neutropenia.^101^ The second patient had a history of Bowen’s disease, recurrent cutaneous warts that became increasingly difficult to treat, as well as cytomegalovirus viremia and colitis.^102^ Impaired expression of ICOSL has also been found in patients with mutations in the *NIK* (NF-*κ*B-inducing kinase)/*MAP3K14*-encoding gene. These patients exhibited B-cell lymphopenia, decreased frequencies of class-switched memory B cells, and hypogammaglobulinemia caused by impaired B-cell survival.^103^ Furthermore, the expression of ICOSL is significantly reduced during the infection of antigen-presenting cells by herpesviruses. For instance, the protein immunoevasin m138/fcr-1 produced by the murine cytomegalovirus (MCMV) was found to physically interact with ICOSL, impeding its maturation and promoting its lysosomal degradation.^102^ This effect of immunoevasin on ICOSL may explain the inadequate T-cell responses and the inability of the host to control virus proliferation during acute MCMV infection. Accordingly, blocking ICOSL in MCMV-infected mice led to a reduction in T follicular helper (Tfh) and GC B cells.^102^ Overall, these findings suggest that the expression of both ICOS and ICOSL is essential for an effective immune surveillance. In line with this, a comparison of samples from patients and mice with breast cancer, both before and after neoadjuvant chemotherapy, identified a subset of ICOSL-expressing B cells that enhance antitumor immunity by increasing the ratio of effector to Tregs.^104^

The binding of ICOSL to ICOS instigates T-cell activation through direct signaling; however, “reverse signaling” (ie, ICOS binding to ICOSL) also induces a signal transduction pathway within ICOSL-expressing cells. For example, the triggering of ICOSL by ICOS-Fc inhibited the adhesion and migration of human umbilical vein endothelial cells cells, whereas the injection of ICOS-Fc in mice reduced the development of lung cancer metastases in the B16 syngeneic melanoma model.^105^^,^^106^ The interaction of ICOSL with ICOS generates various activities among different T-cell subsets, including their activation and effector functions. However, sustained ICOS-ICOSL signaling can lead to suppressive activities mediated by Tregs.^107^ For instance, interactions between ICOSL-expressing melanoma cells and ICOS-expressing CD4+ T cells promote the expansion of immunosuppressive Tregs, enabling immune surveillance escape.^91^^,^^108^ This suggests that ICOS signaling may also be implicated in the termination of the immune response. Additionally, osteopontin, which plays pleiotropic roles in cell adhesion, migration and survival, inflammation, and immune response, can bind to ICOSL through a distinct interacting domain separate from that used by ICOS.^109^ Given that myeloma cells seem to express both ICOSL and ICOS,^110^ the impaired ICOS/ICOSLG signaling reported in multiple myeloma may be attributed to osteopontin binding to ICOSL. Indeed, triggering ICOSL signaling using ICOS-Fc has been shown to inhibit the migration of multiple myeloma cell lines in vitro. Furthermore, the transduction of MOPC-21 cells with a vector overexpressing ICOSL impaired the growth of myeloma tumors following the injection of these cells in immunodeficient NOD scid gamma mice.^110^

After chemical treatment of fibrosarcoma cells, which are highly immunogenic, Sa1N cells give rise to tumors when injected in mice. These cells were then engineered to overexpress ICOSL through retroviral transduction. When injected into syngeneic A/J mice, overexpression of ICOSL resulted in complete tumor rejection.^111^ The destruction of ICOSL-expressing tumor cells by cytotoxic CD8 T cells was remarkably effective, even in the absence of CD4 T cells.^111^ This rejection was attributed to the host immune response to ICOSL-expressing tumor cells, as no effect on tumor growth was recorded when ICOSL-expressing cells were injected into nude mice, which lack a functional immune system.^111^ Furthermore, tumors expressing ICOSL conferred strong antitumor immunity upon rechallenge with the same tumor cells, indicating that the ICOSL signaling pathway may enhance the secondary antitumor response. In a related study, the expression of ICOSL in tumor cells was shown to reduce their tumorigenic potential, making them highly susceptible to destruction by CD8 T cells.^112^ The antitumor activity of ICOSL was further amplified when B16 melanoma tumor cells were first irradiated and then used as a “cellular vaccine” in conjunction with anti-CTLA-4 treatment.^113^ These findings provide robust evidence supporting earlier research that demonstrated the necessity of ICOSL for optimal antitumor responses elicited by anti-CTLA-4 therapy.^114^ Additionally, recombinant ICOSL has been reported to promote antitumor immunity.^115^^,^^116^ Specifically, ICOSL-Fc (also known as B7RP-1-Fc) resulted in tumor rejection or growth inhibition in syngeneic mice, particularly following the injection of highly immunogenic tumor cells.^116^ Moreover, mice injected with these highly immunogenic tumor cells and treated with B7RP-1-Fc exhibited long-lived tumor responses.^116^

High levels of ICOS and ICOSL expression have been significantly associated with improved prognosis in patients with NPC.^117^^,^^118^ In these tumors, the expression of ICOSL was found to be reduced in both lymphatic and distant metastases. Remarkably, the expression of ICOS mirrored that of ICOSL in these tumors, where elevated levels of both proteins correlated with an increased presence of cytotoxic T lymphocytes and higher levels of IFN*γ* in tumor tissues.^117^^,^^118^ Additionally, high levels of ICOS were significantly associated with OS in patients with CRC.^119^ ICOSL activation has been shown to improve the survival of patients with colon cancer,^120^ whereas a chemotherapy-induced shift toward ICOSL-positive B cells in breast tumors was linked to better survival outcomes, likely through enhanced recruitment of cytotoxic T cells.^104^

ICOS activation by ICOSL, however, can stimulate not only T effector cells—specifically CD4(+) Foxp3(−) cells—during anti-OX40-driven tumor immune responses, which facilitate cancer immune rejection, but also Tregs, which promote tumor immune tolerance.^121^ Therefore, an effective strategy for therapies based on ICOS or ICOSL would focus on activating effector T cells, while finding alternative methods to block or reduce ICOS-induced Treg activation.^121^ On the other hand, both ICOSL and HLA-DR (MHCII) are part of a core set of genes characteristic of IgG-positive memory B cells, which possess a significant capacity for proliferation and T-cell interactions but exhibit limited migratory capabilities.^122^ This indicates that ICOSL also plays a crucial role in the adaptive immune response by fine-tuning the IgG-positive memory B-cell response to CD40L stimulation.

## VIII ICOSL reverse signaling

As stated hereabove, ICOS binding to ICOSL has been shown to cause “reverse signaling” that activates a signal transduction pathway within ICOSL-expressing cells. For example, it was found that enhanced ICOSL expression was associated with a worse prognosis in 562 Chinese patients with breast cancer.^123^ In a further study, this group of patients was subdivided into: (1) patients with TNBC, who lacked the expression of estrogen receptor, human epidermal growth factor receptor 2, and progesterone receptor; and (2) the remaining patients with non-TNBC. Database analysis showed that TNBC correlated with the expression of ICOSL, and that ICOSL expression in particular positively correlated with Tregs.^123^ Subsequently, the authors overexpressed ICOSL in MDA-MB-231 breast cancer cells and cocultured them with CD4+ T cells. They found out that the overexpression of ICOSL in MDA-MB-231 cells increased the proportion of FOXP3+ICOS+ Tregs among CD4+ T cells cocultured, as compared with cocultures where MDA-MB-231 did not express ICOSL.^124^ In addition, ICOSL overexpression also increased the secretion of type 2 T helper cell cytokines, including interleukin (IL)-10 and IL-4, by cocultured T cells. The authors further found from experimental data and databases analyses that ICOSL expression was associated with the activation of the p38 MPK pathway and enhanced production of extracellular matrix proteins.^124^ Of note, excessive extracellular protein production correlates with the growth and progression of tumor cells.^125^^,^^126^ These effects of ICOSL were primarily associated with increased phosphorylation of mitogen-activated protein kinase (MAPK) 13/p38delta and MAPK14/p38alpha.^124^ Finally, ICOSL overexpression in MDA-MB-231 enhanced tumor growth upon injection in nude mice.^124^

### A Effects of ICOS-ICOSL in immune checkpoint inhibitor–based anticancer immune therapies

The effects of ICOS/ICOSL signaling pathway in ICI-based anticancer therapies have been the focus of several studies that highlighted its potential to enhance anticancer immunity and improve the outcome of immunotherapies such as those utilizing anti-CTLA-4 or anti-PD-1/PD-L1 antibodies. The significance of the ICOS/ICOSL pathway for optimal anti-CTLA-4-based therapy is underscored by findings that tumor rejection was notably impaired in both *ICOS*- and *ICOSL*-deficient mice.^114^ Moreover, a sustained elevation of ICOS+ CD4 T cells and macrophage survival was noted in the peripheral blood of patients undergoing ipilimumab therapy. This suggests that far from being only a pharmacodynamic biomarker of the response to anti-CTLA-4 therapy, ICOS is a critical target that should be considered alongside anti-CTLA-4 therapy to enhance the effectiveness of ICI-based anticancer treatments.^127^^,^^128^ Additionally, an increased frequency of CD4 + ICOS^hi^ T cells correlated with a higher likelihood of OS.^128^ Supporting this notion, the response to anti-CTLA-4 treatment was significantly diminished in mice with a deficient ICOS/ICOSL pathway compared with wild-type mice.^114^ On the other hand, tumor-infiltrating macrophages play an active role in the shaping of TME. Their transition toward the M1 antitumor phenotype was significantly enhanced when anti-CTLA-4 therapy was combined with an ICOSL-transduced cellular vaccine (IVAX). This combination effectively promoted the migration of T effectors to the TME.^129^ Activating the ICOS pathway using IVAX significantly improved the efficiency of anti-CTLA-4 immunotherapy by generating a robust type 1 T-cell-mediated response.^129^ Consequently, this combination therapy (IVAX combined with anti-CTLA4) resulted in an enrichment in Th1 CD4 T cells and effector CD8 T cells, and in the recruitment and polarization of M1-like antitumor proinflammatory macrophages, driven by IFN*γ* signaling produced by intratumoral CD8 T cells.^129^ Additionally, the intratumoral administration of ICOSL-expressing Newcastle disease virus has been shown to increase the infiltration of activated T cells in both virus-injected and distant tumors, leading to more effective rejection of both tumor types when used in conjunction with anti-CTLA-4 therapy.^130^ This body of evidence strongly supports the idea that harnessing the ICOS-ICOSL signaling pathway, particularly by increasing the expression of ICOSL in tumor cells, can significantly enhance the efficacy of ICI-based therapies in cancer treatment.

On the other hand, in PMEL-1 transgenic mice, which have been engineered to produce CD8+ T cells bearing a TCR specific for the gp100 antigen,^131^ the dual treatment with both anti-CTLA-4 and anti-PD-1 antibodies has been shown to produce a durable antitumor response, particularly by enhancing the effector function of adoptively transferred tumor reactive T cells.^132^ However, deleting Icos in PMEL-1 mice abolished the therapeutic benefits of this dual anti-CTLA-4 and anti-PDL-1 treatment, emphasizing the critical role of ICOS/ICOSL signaling pathway in the antitumor immune response.^132^ Although therapies based on PD-1 blockade enhance the antitumor activity of CD8^+^ T cells, they also inadvertently increase the number of immunosuppressive Tregs, thereby reducing the overall efficacy of immunotherapies against melanoma.^133^ Specifically, anti-PD-1 treatments elevated the numbers of Tregs in mouse models of melanoma, as well as in patients with metastatic melanoma. This increase in Tregs was depended on the production of IL-2 by CD8^+^ T cells, which in turn upregulated ICOS expression in Tregs and facilitated their accumulation within tumors.^133^ To address this issue, the targeting of the ICOS/ICOSL signaling pathway with an anti-ICOSL antibody, aimed at impairing the accumulation of Tregs was explored in combination with anti-PD-1 immunotherapy. The simultaneous blockade of both ICOS and PD-1 signaling pathways did not demonstrate greater efficacy than anti-PD-1 monotherapy. However, a sequential approach, first blocking ICOS followed by blocking PD-1 proved effective in maintaining the number of CD8^+^ T cells while simultaneously reducing the number of Tregs within the tumors. This strategy significantly improved the outcomes of anti-PD-1 therapy.^133^ This approach could provide a viable solution to counteract the problem of anti-PD-1 treatments leading to the accumulation of PD-1-positive Tregs, which can ultimately exacerbate cancer progression.^134^ By strategically targeting the ICOSL signaling pathway, it may be possible to enhance the therapeutic efficacy of existing ICI treatments and improve patient outcomes in cancer immunotherapy.

## IX miR-155 and the regulation of ICOSL expression

Regarding the regulation of ICOSL expression, it is noteworthy that like miR-155, ICOSL is abundantly expressed in B lymphocytes and is essential for the formation of GCs and high-affinity antibody production through T-B cell cross talk. The expression of ICOSL is regulated at the transcriptional level by both canonical and noncanonical NF-*κ*B activities.^135^^,^^136^ The NIK and its upstream BAFF receptor are critical for B-cell expression of ICOSL, which is needed for the development of Tfh T cells.^136^ Notably the injection of a recombinant ICOSL protein into NIK-deficient mice largely rescued their defects in Tfh T-cell development.^136^

At the posttranscriptional level, we recently showed that *ICOSL* transcripts are targets of miR-155, and that the expression of ICOSL is reduced in B cells of E*μ*-*miR-155* transgenic mice as they progressed toward malignancy.^9^ According to Targetscan (Bartel Laboratory; Whitehead Institute for Biomedical Research),^10^ at least 3 human ICOSL isoforms exist within the ICOSL-3′-untranslated region (3′-UTR), including one with a very short 3′-UTR that lacks the *miR-155* binding site. At the posttranslational level, ICOSL can be processed by matrix metalloproteinases ADAM10 and ADAM17, which act as sheddases, resulting in the release of a soluble form of ICOSL.^135^ It is noteworthy that the transcripts coding for ADAM10 and ADAM17 are themselves both predicted targets of miR-155 based on Targetscan software.^10^ Given that there is only one single ICOSL gene and one single gene producing miR-155 primary transcripts per haploid genome, it is likely that the sharp increase in miR-155 levels during the course of the immune response is mirrored by corresponding changes in ICOSL levels.

By targeting both ICOSL and ADAM10 or ADAM17, miR-155 is likely to precisely regulate the intensity of ICOSL signaling. This suggests that the activity of miR-155, by tightly controlling the intensity of ICOS/ICOSL signaling, may fine-tune the intensity and efficiency of the antitumor immune responses. The constitutive expression of ICOSL on naive B cells decreases following B-cell receptor (BCR) engagement by antigens or after treatment with IL-4 or LPS.^89^ In fact, both BCR and toll-like receptor 4 (TLR4) downstream signaling pathways induce miR-155 expression in B cells,^20^, ^21^, ^22^ and *miR-155* global knockout mice exhibit impaired extrafollicular and GC response.^17^ In light of our results, it is probable that the reduction of ICOSL expression following B-cell activation arises, at least in part, from the upregulation of miR-155 after BCR/TLR4 engagement. Interestingly, in addition to the effects of BCR and TLR4 signaling, ICOSL levels are also reduced as a consequence of the binding of ICOS to ICOSL.^137^ This reduction is likely due to a retrocontrol mechanism involving miR-155 and other factors from the ICOS/ICOSL signaling pathway.

## X miR-155 dose effects

Another important parameter to consider is the level at which miR-155 is expressed. In 2 precedent reviews (Tili et al^20^; Michaille et al^22^), we suggested that the facts that miR-155 is present at a single copy per haploid genome and that its level of expression varies greatly during the course of the immune or inflammatory responses^13^^,^^14^^,^^19^ strongly suggest that it may deliver dose-dependent effects. In effect, it has been established that the I*κ*B/NF-*κ*B signaling module controls the temporal activation of genes implicated in the immune response through successive waves of NF-*κ*B nuclear activity.^138^ In fact, at least 2 groups of genes are successively activated then repressed during the course of the immune response,^139^ the first group of genes being needed for the initiation of the response, and genes of the second group implicated in containing the intensity of and terminating the response to avoid deleterious effects to the body. As miR-155 levels rise sharply from the initiation of the response up to its termination, it can be predicted that initial low levels of miR-155 should lead to the targeting of transcripts belonging to the first group, and higher levels of miR-155 found later should allow for the targeting of transcripts from the second group. Indeed, in mouse RAW264.7 macrophages challenged by LPS, we previously found out that low levels of miR-155 caused the targeting of QKI transcripts; however, the subsequent rise of miR-155 levels was associated with increasing levels of Qki transcripts.^19^ Accordingly, in RAW-264.7 cells, an antisense miR-155 inhibitory RNA increased the levels of Qki expression at 4 hours following LPS challenge, but not later.^19^ These results were subsequently confirmed and extended by a study showing that high and intermediate levels of miR-155 expression in acute myeloid leukemia cell lines were functionally distinct, with highly significant differences in targets and downstream genes regulation.^62^ More specifically, high miR-155 levels correlated with increased expression of genes implicated in inflammatory pathways and decreased expression of genes playing a role in cell proliferation, whereas intermediate levels of miR-155 were targeting genes involved in myeloid cell differentiation.^62^ This particular feature of miR-155 activity most probably explains why it has been described as both a protumor and an antitumor microRNA, in relation with its different levels of expression in particular tumors. Of note, when comparing the 3′-UTR of the same gene in human and mouse, it is very clear that human transcripts in general contain many more microRNA target sites than their mouse counterpart, suggesting that microRNA regulation has been progressively extended during the evolution of the primates, most probably in relation with their extended expectation of life and increased necessity to keep the critical gene networks under tight control.

### A miR-155 as both a protumor and an antitumor microRNA

Because of its dual pro- or antitumor activity and its widespread expression, miR-155 is expected to impact all aspects of the antitumor response, either positively or negatively, well beyond its effects on ICIs. In addition, one or more consensus target sites for miR-155 are present in 3297 human transcripts (targetscan.org),^10^ split in a total of 596 sites conserved across species and 3697 poorly conserved sites. Thus, when miR-155 is expressed in the wrong place at the wrong time and/or at an abnormal level, this microRNA can be anticipated to have deleterious effects through multiple gene pathways. Of note, miR-155 expression in the right tissue at the right time and at normal levels is expected to be beneficial, as no “bad gene” can possibly remain in the genome after a long evolutionary process. Here after, we will present a few examples of potential deleterious effects of miR-155 according to its proven or putative targets.

## XI miR-155 and the p38*α* pathway

The p38*α* can be activated by MAPK kinases MKK3 and MKK6 (reviewed by Mittelstadt et al^140^; Cuenda and Rousseau^141^). It is very abundant in most of the cell types, and is widely implicated in inflammatory diseases, through it control of the production of inflammatory cytokines and chemokines by cells of the innate and the adaptive immune systems.^142^, ^143^, ^144^, ^145^ The p38*α* has also pleiotropic effects in cancer through its effects on cell proliferation, differentiation, and survival (reviewed by Wagner and Nebreda^146^; Gupta and Nebreda^147^). Interestingly, the isoform of the p38*α* 3′-UTR that is 9924 nt in length as well as the 3′-UTR of MKK6 respectively contain 1 and 2 miR-155 target sites. The inactivation of the mouse *Ppm1d* gene, which codes for the Wip1 phosphatase, leads to the activation of the tumor suppressor TP53/p53 and cyclin dependent kinase inhibitor 2A (CDKN2A)/p16/INK4A through the p38 MAPK.^148^ Thus, *Ppm1d* inactivation impaired the transformation of mouse embryo fibroblasts by oncogenes. In vivo, *Ppm1d* inactivation impaired the development of mammary tumors in mice overexpressing either the Erbb2 or Hras1 oncogene under a mouse mammary tumor virus promoter, an effect that was suppressed by inactivating the gene coding for p38*α*.^148^ Of note, the 3′-UTR of the human *PPM1D* gene also contains a miR-155 target site, suggesting that all this regulatory pathway may be impacted by variations of miR-155 levels.

On the other hand, p38*α* has been reported to have protumor effects, and increased levels of phosphorylated p38*α* have been found in different cancers, including lung, thyroid, and breast carcinomas, as well as in head and neck squamous cell carcinomas, gliomas, and follicular lymphomas (FLs).^149^, ^150^, ^151^, ^152^, ^153^, ^154^ For example, it has been shown in a mouse model of breast cancer that the killing of tumor cells by the DNA damage-inducing molecule cisplatin increases upon inhibition of the p38*α* MAPK pathway.^155^ This was linked to the reactive oxygen species–dependent increased phosphorylation of both JNK1 and JNK2 MAP kinases, whose proproliferative activity resulted in more cisplatin-induced DNA damage followed by apoptosis.^155^ Thus, in the context of cisplatin treatment, p38*α* activity favors the proliferation of cancer cells, at least in this setting. Accordingly, several lung tumors presented with increased p38 MAPK phosphorylation, although it was not clear how this MAPK would increase lung tumorigenesis in this context.^149^ On the other hand, the simultaneous inhibition of the p38*α* and the MEK/ERK pathways induces apoptosis both in vitro and in preclinical mouse models.^156^ In a study involving 316 patients with CRC in stages I-III, the patients from the subgroup with high levels of phosphorylated p38*α* had the lowest survival probability, and phosphorylated p38*α* was a significantly independent factor for death, recurrence, and distant metastases.^157^ In another study, authors used clones derived from HCT-116 and SW480 cell lines that were rendered resistant to SN38 (the active metabolite of the irinotecan molecule used in chemotherapy) to investigate the role of the p38 pathway in the resistance to SN38. They found that treating the cells with SB202190, an inhibitor of both p38*α* and p38*β*, enhanced the cytotoxic activity of SN38.^158^ The same authors further showed that p38 inhibition sensitizes tumor cells derived from both SN38-sensitive and SN38-resistant HCT116 cells to irinotecan treatment in xenograft models, and also that primary colon cancer of patients sensitive to irinotecan-based treatment presented with less phosphorylated p38 than that of nonresponder patients.^158^

Thus, considering that p38*α* may inhibit the development of certain tumors while working as a tumor promoter in other cancers, any strategy based on targeting the p38*α* pathway should be very carefully designed to avoid unwanted results. Also, given the potential targeting of MKK6 and p38*α* by miR-155, any treatment based on the modification of miR-155 expression should be adjusted to avoid harming patients through miR-155 effects on the activity of the p38*α* pathway.

## XII miR-155 and the JNK pathway

Another MAPK pathway, the c-JNK pathway, has been implicated in cancer cell survival or apoptosis, depending on the type of stimuli and the strength and duration of their activation (reviewed by Wu et al).^159^ The JNK family contains members MAPK8/JNK1, MAPK9/JNK2, and MAPK10/JNK3. The main target proteins are the members of the JUN family of AP1 transcription factors. These transcription factors regulate a number of genes whose promoter contains an AP1 binding site, which are generally implicated in the control of the cell cycle, survival and apoptosis, and metalloproteases.^159^ JNK1 and JNK2 usually exert opposite effects: thus, JNK1 increases cell survival, whereas JNK2 mediates apoptosis and cell death. Thus, the expression of the essential transcription initiator Tata-binding protein is upregulated by JNK1 but downregulated by JNK2, which in both cases serves for the regulation of the expression of the c-Jun AP-1 factor and of fibroblast proliferation rate.^160^ On the other hand, JNK1 alone is sufficient for TNF-induced apoptosis of mouse fibroblast.^161^ In a study on different epithelial cancer cell lines including human colorectal carcinoma, HCT116, breast carcinoma, and osteosarcoma, the regulation of cancer cell survival was found dependent on the opposing functions of the prosurvival JNK2/Bcl-3 pathway and the proapoptotic JNK1/c-Jun pathway.^162^ Although less studied than JNK1 and JNK2, JNK3 has been involved in prostate cancer. Thus, JNK3 increases the expression of EZH2, an oncogene playing a key role in prostate cancer. Although high levels of JNK3 and EZH2 correlate with patients with prostate cancer with a worse prognosis, the downregulation of JNK3 inhibited proliferation and invasion of cancer cell lines, and in contrast promoted apoptosis.^163^ One way for tumor cells to escape immune surveillance is through the secretion of transforming growth factor beta (TGF*β*), a cytokine that impairs the expression of IFN*γ* by cytotoxic T cells.^164^ Following injection of U87 glioma cells in zebrafish embryos, TGF*β*1 signaling enhanced angiogenesis. This enhancement was specifically blocked by a JNK inhibitor, but not by inhibitors of p38 MAPK, ERK MAPK, or PI3K.^165^ Of note, the 3′-UTRs of transcripts encoding SMAD2 and SMAD3, both implicated in the TGF*β* signal transduction to the nucleus, contain a putative miR-155 target site, as do the 3′-UTRs of JNK1 and JNK3 transcripts.

## XIII miR-155 and the regulation of apoptosis

Apoptosis plays a key role in the maintenance of tissue homeostasis. This process of programmed cell death depends on the activation of a cascade of proteolytic enzymes called caspases. Apoptosis can occur through the extrinsic or intrinsic (ie, the mitochondrial) pathways.^166^^,^^167^ The key enzyme is the cysteine-aspartic acid protease caspase 3 (CASP3), which is produced as a pro-caspase and then activated through proteolysis.^166^, ^167^, ^168^ When a cell is damaged, a cascade of reactions leads to DNA fragmentation, degradation of the cytoskeleton and nuclear proteins, expression of ligand for phagocytes, and ultimately formation of apoptotic bodies.^167^^,^^169^^,^^170^ In the intrinsic pathway, the pro-caspase-9 becomes able to bind the adapter protein apoptotic protease activating factor 1 (APAF1), leading to the opening of the mitochondrial permeability transition pore and the release of cytochrome C and other proteins. Depending on the cell type, the release of the cytochrome C is regulated by a delicate balance between pro-apoptotic—the activators PUMA, BID, and BIM, and the effectors BAX and BAK—and antiapoptotic—BCL2, BCLW, BCLXL, MCL1, and BFL1—factors of the BCL2 family.^171^^,^^172^ The binding of cytochrome C to APAF1 monomers causes several APAF1s to associate and form the apoptosome, which then recruits and activates several pro-caspase-9. The activated caspase 9 then activates the pro-caspase-3,^173^ producing the active caspase-3 that ultimately induces apoptosis.^166^, ^167^, ^168^^,^^174^ JNK1 and JNK2 activities can elicit a survival response in fibroblasts treated with TNF. This effect is mediated by the transcription factor JunD, which can collaborate with NF-*κ*B in increasing the expression of prosurvival genes, including the gene coding for the Inhibitor of apoptosis protein cIAP-2/BIRC3, whose 3′-UTR contains a *miR-155* binding site.^175^ On the other hand, following LPS challenge of mouse RAW 264.7 macrophages, the activation of the TLR4 pathway initiates the targeting of *CASP3* transcripts by *miR-155*, thus impairing apoptosis. This effect can be reversed by treating the cells with an anti-miR-155 antagomir.^176^ Accordingly, in patients with thyroid follicular carcinoma, the expression of miR-155 was negatively correlated with the expression level of CASP3.^177^ Remarkably, although BCL2 transcripts contain a miR-155 target site, there was a positive correlation between miR-155 levels and BCL2 levels in the same patients,^177^ which provides further evidence of miR-155 having dose-dependent effects. More recently, it has been established that CASP3 plays additional roles in pyroptosis and necrosis (reviewed by Qi et al^178^). In the first described type of pyroptosis, inflammatory signals cause inflammatory caspases 1, 4, 5, and 11 (cf. the review by Sahoo et al^168^) to cleave gasdermin G to generate a carboxy-terminally truncated peptide that creates holes in the plasma membrane.^179^, ^180^, ^181^, ^182^ In a second type of pyroptosis, however, TNF or chemotherapy drugs activate CASP3, causing cleavage of gasdermin E followed by formation of holes in the cell membrane and pyroptosis,^183^^,^^184^ a process that was possible with the intervention of either BAK or BAX factor alone.^185^ Accordingly, miR-155 was shown to inhibit Casp3 and phosphatase and tensin homolog (PTEN) activity and to increase the phosphorylation of the PI3K/AKT, thus promoting PI3K/AKT activity and proliferation of the NPC cells CNE2.^186^ On the other hand, although CASP6, originally described a pro-apoptotic executioner caspase, has not been as extensively studied as the other caspases, it was shown that resveratrol, a polyphenol with a wide range of anti-inflammatory, antiproliferation, and anticancer effects,^187^ induces apoptosis of HCT116 colon cancer cells through a mechanism that implicates the cleavage of Lamin A by CASP6 (whose 3′-UTR contains a *miR-155* consensus target site) and that remains functional even in the absence of Bax or p53 activities.^188^ It was later established that resveratrol also decreases miR-155 expression in particular by decreasing the levels of AP-1 factors JunB and Jun D.^189^

In conclusion, miR-155 effects on cell death pathways such as apoptosis, pyroptosis, and necrosis, as well as on cell proliferation, are potentially crucial for cell homeostasis and tumor induction and development, considering that the 3′-UTR of the genes encoding APAF1, the caspases 3, 6, and 9, the apoptotic activator BID, the apoptotic effector BAK, the antiapoptotic factors BCL2 and BCLW, the inhibitor of apoptosis cIAP-2/BIRC3, the CDKN1B/p27KIP1, and the phosphatase PTEN (that inhibits the proproliferation PI3K/AKT pathway by dephosphorylating PIP3 back into PIP2) all contains a consensus target site for miR-155. Given the opposite roles of the above factors on cell death and proliferation, it can be expected miR-155 activity to alternatively favor cell homeostasis or cell proliferation depending on its level of expression.

## XIV Epigenetic changes

The accession of transcription factors and the associated cofactors to their target promoters is regulated by the chromatin-remodeling complex SWItch/Sucrose Non-Fermentable (SWI/SNF). Key to the activity of this complex are factors with an ATP activity, namely BRM/SMARCA2 (SWI/SNF related BAF chromatin remodeling complex subunit ATPase 2) or BRG1/SMARCA4. Aberrant BRG1 expression has been found in advanced stages of gastric,^190^ prostate,^191^ melanoma,^192^ and non–small cell lung cancer,^193^ suggesting a tumor-suppressor role for BRG1 in these types of tumors. In contrast, BRG1 plays a key role in leukemia maintenance, and leukemic cells that lack BRG1 activity quickly undergo cell-cycle arrest and apoptosis.^194^, ^195^, ^196^ The 3′-UTR of *BRG1* transcripts contains a miR-155 consensus target site, and an inverse correlation between BRG1 and miR-155 expression has been found in Burkitt lymphomas (BLs) and DLBCLs.^197^ Therefore, through the regulation of BRG1 and potentially other epigenetic regulators, miR-155 effects could potentially impact the expression of a number of factors implicated in cancer whose transcripts do not contain miR-155 target sites.

## XV Exosomes

Independent of its expression or lack of expression in a given cell type, miR-155 can pass from one cell to another through exosomes. For example, ischemic, hypoxic, obstructive, metabolic, and especially inflammatory injury play a key role in inducing renal tubular epithelial cell senescence that can cause chronic kidney disease.^198^, ^199^, ^200^, ^201^, ^202^, ^203^ Similarly, cisplatin, a molecule that causes DNA damage usually followed by cell death, nevertheless can often cause tubular epithelial cell injury leading to acute kidney injury.^204^ One culprit was established to be miR-155, as impairing its expression reduced apoptosis, genome instability, and telomeric dysfunction of tubular epithelial cells, at least in part through the increase of telomeric repeat binding factor (TERF1/TRF1) and cyclin dependent kinase 2.^204^ Of note, the 3′-UTR of the transcripts encoding these 2 factors both contain a miR-155 consensus target site. Interestingly, it was further demonstrated that miR-155-containing exosomes released from macrophages were internalized by tubular epithelial cells where miR-155 causes telomere shortening and malfunction through targeting TERF1 transcripts.^205^

On the other hand, it was shown that miR-155 transferred from oral squamous cells resistant to cisplatin to similar cells sensitive to cisplatin induces their epithelial-to-mesenchyme transition and enhanced their migratory capability and their resistance to cisplatin.^206^ It was also found that, following treatment with the anticancer drug gemcitabine, the levels of miR-155 increased in both pancreatic ductal adenocarcinoma cells and exosomes.^207^ Increased miR-155 expression after long-term gemcitabine treatment caused exosome secretion and resistance through the activation of antiapoptotic activity, with exosomes subsequently increasing miR-155 levels in other pancreatic ductal adenocarcinoma cells and spreading resistance.^207^ In vitro, the resistance of pancreatic cancer cells to gemcitabine treatment was shown to be caused by exosome-delivered miR-155, followed by the downregulation of deoxycytidine kinase, an enzyme needed to produce the monophosphate form of gemcitabine, a step critical for its subsequent transformation into its triphosphate active form.^208^ Of note, the 3′-UTR of deoxycytidine kinase contains a consensus miR-155 target site. Along the same line, exosomes derived from a gastric cancer cell line resistant to paclitaxel (MGC-803R cells), a first-line chemotherapeutic agent for gastric cancer, were enriched with miR-155.^209^ The treatment of MGC-803R cells with miR-155-enriched exosomes as well as miR-155 overexpression in these cells induced a malignant phenotype of sensitive cells, at least in part through miR-155 directly target the 3′-UTR of the GATA binding protein 3 (GATA3) transcription factor and to that of tumor protein p53-inducible nuclear protein 1 (TP53INP1).^209^ Similarly, in breast cancer, miR-155 expression was increased in epithelial cells undergoing epithelial-to-mesenchyme transition. Exosomes derived from cancer stem cells as well as of cells resistant to chemotherapy showed enhanced concentration of miR-155, which were able to transmit resistance and migration capacities to cells sensitive to treatment.^210^ Finally, the possibility of miR-155 passage form one cell to another through exosomes adds another layer of complexity of regulation of the dose-dependent effects of this microRNA.

## XVI Lymphomas

Within lymphoid follicles, GCs represent the location where B cells are progressively selected for their increasing affinity to antigens.^211^, ^212^, ^213^, ^214^ In the GC dark zone (DZ), B cells proliferate and undergo somatic hypermutations and class switching, afterward they migrate toward the light zone (LZ), where they encounter antigens presented to them by follicular dendritic cells and are selected, through their interactions with Tfh cells based on their affinity for antigens.^211^, ^212^, ^213^ A few selected cells then proliferate and differentiate into antibody-secreting plasma cells or memory B cells; however, some return to the DZ to sustain a new cycle of hypermutation and proliferation. Thus, through successive cycles, plasma cells produce antibodies of increasing specificity for the antigen presented by Tfh cells.^211^ The formation and proper functioning of the DZ is regulated by the FOXO1 transcription factor activating the transcription of the gene encoding the CXCR4 chemokine receptor and cooperating with the transcription factor BCL6 to impair the expression of genes implicated in immune activation, DNA repair, and differentiation of plasma cells.^215^ On the other hand, the behavior of B cells in the LZ is controlled by the PI3 kinase signaling pathway that downregulates FOXO1 expression.^216^ As a consequence of the lessening of the activity of pathways implicated in DNA damage repair and proliferation checkpoints (in order to allow cells bearing new mutations to further survive and proliferate), the GC zone constitutes a potential hot bed for malignant transformation because of off-target mutations.^211^ Indeed, B cells passing through the GC reaction are responsible for the majority of non-Hodgkin lymphomas that present with characteristics reminiscent of those of GCs. Those are in the decreasing order of frequency: (1) DLBCLs, aggressive and rapidly evolving; (2) FLs, a most usually indolent tumor of the old age; and (3) BLs, mostly found in children and young adults.^211^ One can distinguish 2 main subgroups of DLBCLs: the GC B cell-like (GCB-) DLBCLs,^217^ together with FLs, whose transcriptome resembles that of B cells from the LZ of GCs,^218^ and the activated B cell-like (ABC-) DLBCLs, whose transcriptome resembles that of plasmablasts.^219^^,^^220^ We recently found a negative correlation between the levels of miR-155 expression and those of both ICOSL and MHC-I, with evidence of dose-dependent effects at low miR-155 levels in both cases.^221^ In agreement with the above classification, the reduction of ICOSL expression was associated miR-155 targeting *ICOSL* transcripts in ABC, however not in GCB, primary tumors, and cell lines. Our results suggested that miR-155 downregulation of MHC-I in DLBCLs and of ICOSL at least in GCB DLBCLs were primarily indirect, either caused by inhibition of mRNA translation or the targeting of some of their positive regulators.^221^ Of note, this dual inhibition of ICOSL and MHC-I was also found in NPCs classified as types 2 and 3 by World Health Organization,^222^ which suggests that it could represent a common feature among both solid and hematological malignancies. Types 2 and 3 NPCs are characterized by poorly differentiated carcinoma cells and are strongly associated with Epstein–Barr virus infection.^223^ Strikingly, Epstein–Barr virus-infected cells show upregulation of both miR-155 and its target PD-L1, against suggestive of miR-155 delivering dose-dependent effects.^222^ It should further be noted that, by downregulating both MHC-I and ICOSL, miR-155 renders tumor cells invisible to tumor-invading T lymphocytes, making them frustrated by blocking their activation and cytotoxic activity. Of note, the lack of presentation of tumor-specific antigens by MHC-I is normally expected to initiate the antitumoral response of NK cells. However, miR-155 also can potentially target transcripts encoding CD58/LFA-3, a membrane protein that is needed for the adhesion and activation of both T lymphocytes and NK cells.^224^

Among the pathways implicated in B-cell proliferation and selection that are likely to be impacted by miR-155 activity for the process of selection and amplification of B cells with the greatest possible affinity for the antigen to take place rapidly, different pathways implicated in the maintenance of DNA integrity and the control of cell proliferation have to be bypassed at least in part.^211^ This to allow among others: (1) the action of various tumor suppressor factors, including CDKN1A/p21CIP1, CDKN1B/p27KIP1, and TP53^225^^,^^226^; (2) the activation induced cytidine deaminase (AICDA/AID) enzyme that produces somatic hypermutations, however, also potentially producing off-target mutations.^227^ Of note, the 3′-UTRs of *CDKN1B* and *AICDA* both contain a miR-155 consensus target site. In addition, among the naïve B cells that move to the B-T border to interact with CD4+ Th following antigen encounter, the most specific receive stimulatory proproliferation signals before migrating to the center of the follicle to begin their initial expansion in order to form a new GC.^228^ At that moment, any initial lesion affecting the sequence of the transcripts encoding the Tet methylcytosine dioxygenase 2 (TET2) in hematopoietic stem cells or the inhibitor of apoptosis *BCL2* in pre-B cells will give the bearer a survival and proliferative advantage.^229^, ^230^, ^231^ Of note, both the TET2 (5279 nt isoform) and BCL2 transcripts contain a miR-155 consensus target site. BCL6 normally represses *NOTHCH2*, whose transcripts contain a miR-155 consensus target site, in resting B cells within the GC. NOTCH2 expression in B cells suppresses GC formation, and in contrast it induces marginal zone differentiation and inhibits the growth of GC-derived lymphoma cells.^232^ About 49% of patients suffering from EZB DLBCLs (associated with EZH2 mutations and BCL2 translocations),^233^ which presents with mutations of the NF-*κ*B and JAK/STAT pathways,^211^ carry amplification or activating mutations of STAT6 or an inactivating mutations of its negative regulator SOCS1, which favors survival and proliferation of lymphoma cells.^233^ Here again, *SOCS1* 3′-UTR contains a miR-155 target site. B cells homing in the GC rests on S1PR2 and GNA13 activities. Thus, the sphingosine-1-phosphate (S1P) inhibits the migration of GC cells through its receptor S1PR2 and the G-protein GNA13.^234^ As a consequence of the lack of GNA13 activity, the confinement of B cells within the GC B cells is lost, causing the dissemination of lymphomas originating from the GC.^235^ Indeed, *GNA13* mutations are found in 30% of GCB-DLBCL^236^ and 15% of BL.^233^^,^^237^ A similar effect can be expected from increased *miR-155* activity, given the presence of its target sequence in *GNA13* 3′-UTR. The gene encoding SMAD family member 1, whose 3′-UTR contains a miR-155 target site, is involved in the bone morphogenic protein pathway. Its silencing through hypermethylation causes DLBCL resistance to chemotherapy that can however be reversed using DNA methyltransferase inhibitors that work well in combination with chemotherapy.^238^ The para-caspase MALT1, a critical mediator of the BCR signaling pathway, is instrumental in the development of ABC-DLBCL, mantle cell lymphoma, primary effusion lymphoma, and CLL.^239^, ^240^, ^241^, ^242^, ^243^, ^244^ Inhibitors of MALT1 impair the development of ABC-DLBCLs both in vitro and in vivo,^245^, ^246^, ^247^, ^248^ and are also effective in CLL patients, even those who developed ibrutinib resistance.^244^ Again, the 3′-UTR of *MALT1* transcripts contains a miR-155 target site, making it a potential target of this microRNA.

In the LZ of the GC, B-cell selection takes place through antigen recognition on follicular dendritic cells by the BCR and by antigen presentation to CD4+ Tfh cells via the MHC II. The engagement of the costimulatory CD40 receptor on B cells by its ligand (CD40L) on CD4+ Tfh cells stimulates expression of MHC II genes as well as of adhesion molecules like ICAM-1 and CD58.^249^ Of note, CD58 3′-UTR isoform 279 nt in length contains a miR-155 target site, placing the stability of B-cell attachment to Tfh cells under *miR-155* control, especially given the effects of this microRNA on ICOSL (see hereabove). In the same way, important components of the immune synapse such as PD-L1 and CD47, with both a 3′-UTR containing a *miR-155* target site, can be suppressed to exacerbate the immune tolerance privileges of B cells.^250^, ^251^, ^252^ Also present on Tfh cells, the B- and T-lymphocyte attenuator, with a 3′-UTR bearing a miR-155 target site, limits GC development through its binding to the TNF receptor TNFRSF14 and its reduction of IL-21 expression and is often deleted in patients with FL and DLBCL.^253^ Accordingly, TNFRSF14 knockdown accelerated tumor development in a mouse model of BCL2-driven lymphoma.^254^

Finally, other genes implicated in the maintenance of DNA integrity have to be downregulated to allow for the rapid proliferation of selected B cells, and plasmablasts produced are not supposed to normally present an extended life. Among these genes, several can potentially be impacted by *miR-155* activity, because of the presence of a *miR-155* consensus target site in their 3′-UTR: the mismatch repair factors MutS homolog 2 (MSH2), MutS homolog 6 (MSH6), and MutL homolog 1,^61^ as well as the G2 checkpoint kinase Wee1.^55^

## XVII Conclusion

**Fig. 2:**
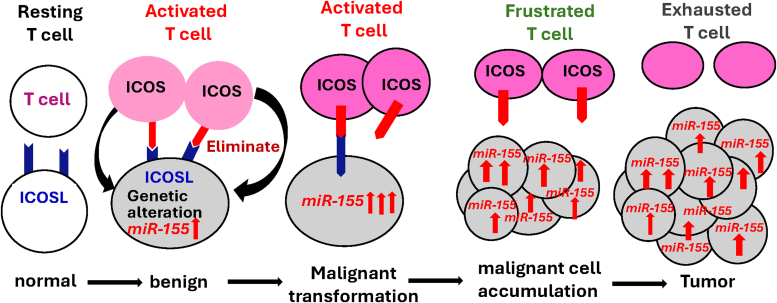
A stepwise model illustrating an exacerbated miR-155 activity causes loss of ICOSL expression and decreases the efficacy of antitumor immune response. Initially, in healthy cells, miR-155 is absent. As a consequence of various genomic alternations cells can start to express miR-155. At this stage of cellular transformation, tumor cells are still recognized and eliminated by T cells through TCR-MHC (not shown) and ICOS-ICOSL interactions. However, if further genomic alternations elevate miR-155 levels, the expression of ICOSL by tumor cells decreases significantly. This loss hinders activated T cells, which express ICOS, from effectively recognizing and binding to the tumor cells, producing frustrated T cells. Over time, this frustration results in T-cell exhaustion, marked by reduced responsiveness and diminished cytotoxic activity. Meanwhile, malignant cells with high miR-155 levels and no ICOSL continue to proliferate, fully evading immune detection. This interplay between miR-155 activity, ICOSL loss, and T-cell exhaustion represents a crucial mechanism in tumor immune evasion.

Our model of E*μ*-*miR-155* transgenic mice, where the dysregulation of a single microRNA results in an aggressive B-cell malignancy at the penetrance of 100%, allowed us to discover that such mice do not express ICOSL, a target of miR-155, on the surface of the malignant cells.^9^ In contrast, switching the transgene off resulted in the re-expression of ICOSL on the surface of cancer cells, followed by activation and expansion of cytotoxic T cells, and killing of the malignant cells (Fig. 2). The simplicity of this model underscores the fact that ICOSL plays a major role in the control of tumor rejection. Therefore, miR-155 regulation of ICOSL but also other immune checkpoints can significantly influence the effectiveness of immunotherapies. For example, *miR-155* targeting of CTLA-4 may enhance T-cell activation, which can be advantageous in therapeutic settings. Conversely, the modulation of ICOSL by miR-155, as described here, can hinder T-cell recognition and killing of tumor cells. It is thus probable that both the levels and timing of miR-155 expression are critical for optimal response to immunotherapies. Understanding the regulatory network involving miR-155 and immune checkpoints such as CTLA-4,^30^ PD-1/PD-L1,^255^ and ICOSL,^9^ is thus essential for developing strategies to improve the efficacy of immunotherapies of various cancers. By manipulating miR-155 levels or its targets, it may be possible to fine-tune the immune response and enhance tumor recognition and elimination. Particularly, ICOSL-based therapies could significantly improve the treatment of malignancies characterized by high levels of miR-155, not only of hematological cancers, but also of common solid tumors such as breast, lung, and colon cancer, where elevated levels of this microRNA have been reported.^37^ Additionally, other microRNAs that are dysregulated in cancer are anticipated to influence the expression and activity of various immune checkpoints, similar to the effects of miR-155 on ICOSL. Studying the interactions between these microRNAs and immune checkpoints is expected to yield new insights and identify novel therapeutic approaches.

It should not be disregarded, however, that a number of other microRNAs that have been described as either protumor or antitumor can have a significant impact on the antitumor immune response for both hematological and solid cancers either through canonical or noncanonical functions.^256^^,^^257^ Among them, several can target transcripts encoding CTLA-4, PDL1, or ICOSL.^258^^,^^259^ As the expression levels of these microRNAs, as well as that of miR-155, can change during the different stages of tumors, and given that these microRNAs can also influence each other’s expression, the manipulation of the levels and activity of these pleiotropic regulators in the treatment of cancers will be rather delicate.

Indeed, although a lot of promising results have been drawn from studies on cell cultures, no convincing results have been obtained to date in human, and trials conducted to test the use of microRNA agonists or inhibitors have been stopped in clinical phase I or II.^260^ In the case of miR-155, a clinical trial (NCT03837457) based on the use of cobomarsen (also known as MRG-106) that is a locked nucleic acid-based miR-155 inhibitor has been conducted to investigate the efficacy and safety of this molecule in subjects with cutaneous T-cell lymphoma, mycosis fungoides subtype has been terminated early in clinical phase II. Thus, at least in the near future, it is more than probable that microRNAs will be used as biomarkers of cancer development and evolution rather as therapeutic agents.

Another complication comes from the fact that the set of mRNA targets for a given miRNA can vary during the course of tumor evolution, in particular through differential polyadenylation, with consequences on cell proliferation and differentiation, as well as on the process of cell transformation.^261^ It has thus been found that, depending on the cellular context, transcripts can use shorter 3′-UTRs, which decreases the number of target sites for different miRNAs.^262^ Thus, it has been found that, although activated CD4+ helper T lymphocytes indeed use shorter 3'-UTRs,^263^ however the consequences of 3′-UTR shortening on mRNA and protein accumulation remain rather limited.^264^ More recently, however, a remarkable study by Hsin et al^265^ has shed a new light on how miR-155 can exert cell- and context-dependent effects on the same 3ʹ-UTR in mouse B cells, CD4+ T helper lymphocytes, dendritic cells, and activated macrophages. Their comparative analysis of miR-155-bound sites showed that, although miR-155 sites shared across macrophages, dendritic cells, and CD4+ T and B cells tend to exhibit more extensive seed complementarity, miR-155-bound sites shared by 3 or less cell types tend to have reduced seed matches. In addition, a significant percentage of the 3′-UTRs of miR-155 sites lacking a hexamer-seed match corresponded to cell-type restricted transcripts, yet several mRNAs with noncanonical miR-155 sites were nevertheless significantly repressed.^265^ Furthermore, cells with the highest levels of miR-155 expression showed the greatest number of miR-155 binding sites, a further illustration of the capability of miR-155 to deliver dose-dependent effects.^265^ Interestingly, although CD4+ T cells showed that the 3′-UTRs of mRNAs did exhibit a tendency toward shortening in proliferating cells, many genes turned to producing longer 3'-UTR isoforms that contain a higher number of miR-155-binding sites, placing them under tighter miR-155-mediated regulation.^265^

Well beyond ICOSL, owing to the number of potential target transcripts (targetscan.org), miR-155 can regulate multiple pathways involved not only in the inflammatory and the immune responses, but also in cell apoptosis, proliferation, and homeostasis. We thus cannot be surprised by the number of molecular pathways impacted by modifications of the level of expression of this microRNA, either in normal or pathological situations. Of note, *miR-155* seed sequence (UAAUGCU), as well as its variants, usually contains only 2 G/C nucleotides. It should also be noted that miR-155 target sequences generally map within 3′-UTRs rich in A/T nucleotides. Globally, miR-155 target genes usually seem to map in isochores poor in C/G nucleotides, which can be surprising given that this microRNA is often called into action in situations of inflammation and/or infection that are associated with fever. The fact that the primer melting temperatures (Tm) and thus the stability of pairing of sequences rich in A/T are lower than those of sequences rich in G/C within mammalian genomes where coding regions are more frequently found in isochores rich in G/C, with 34% of the genes being located in the GC-poor isochores (which represent 62% of the genome), 38% in the GC-rich isochores (31% of the genome), and 28% in the GC-richest isochores (3% of the genome).^266^ This suggests an adaptation that could possibly translate into *miR-155* targeting transcripts that encode inflammatory and immune factors in a temperature-dependent manner, thus increasing the velocity of the mounting of inflammatory and immune responses. As *miR-155* nevertheless is supposed to take over at the time of termination of the response, the necessity to decrease the value of the constant of dissociation (Kd) at this time (ie, to increase the stability of *miR-155* binding to its target transcripts) may possibly explain why the range of expression of this microRNA is so large, with a huge increase of its level of expression during the course of the inflammatory or immune response. Furthermore, AU-rich elements (AREs) are 30 UTR cis-regulatory elements that regulate the stability of mRNAs.^267^^,^^268^ Through their interaction with different RNA-binding proteins, they can facilitate or impair the degradation of ARE-containing transcripts.^269^ Interestingly, mature human miR-16 contains an UAAAUAUU motive complementary to the ARE sequence. miR-16 plays a role in ARE-RNA decay through in cooperation with tristetraprolin, a protein that associates with Ago/eiF2C family members and assists in the targeting of ARE without directly binding to miR-16,^270^ a mechanism that also could possibly be influenced by the temperature. Finally, it is probable that miR-155 could also participate in the targeting of ARE-containing transcripts through interaction with tristetraprolin or other RNA-binding proteins, given that it contains a sequence similar, although not identical, to ARE, that would thus represent a noncanonical binding site as described by Jing et al.^270^

In conclusion, the above considerations establish *miR-155*, a microRNA newly arose during the course of vertebrate evolution with its many putative targets (the ones cited in the main text being given in Table 1), as a master regulator of the response to cellular and environmental dangers.

**Table 1:** Human genes whose 3′-UTR contain a miR-155 consensus target site, making their expression and function potentially vulnerable to miR-155 abnormal expression

Genes (Indicated Isoforms Are for the 3′-UTR)	Encoded Proteins	Main Functions
*ADAM10*	ADAM10	ADAM metallopeptidase domain 10/cell surface protein possessing both potential adhesion and protease domain/act as sheddase for ICOSL
*ADAM17 (1718 nt isoform)*	ADAM17	ADAM metallopeptidase domain 17/cell surface protein possessing both potential adhesion and protease domain/act as sheddase for ICOSL and for TNF
*AICDA*	AICDA/AID	Activation induced cytidine deaminase (somatic hypermutation and class-switch recombination)
*APAF1*	APAF1	Apoptotic peptidase activating factor 1/allows the opening of the mitochondrial permeability transition pore (intrinsic pathway)
*BAK*	BAK1	Apoptotic effector
*BCL2 (5279 nt isoform)*	BCL2	Apoptosis regulator/outer mitochondrial membrane protein that blocks the apoptotic death of some cells such as lymphocytes
*BCLW*	BCL2L2	Anti-apoptotic factor
*BID*	BID	Truncated form (tBID) is an apoptotic activator
*BIRC3*	cIAP-2	Baculoviral IAP repeat containing 3 (inhibits apoptosis by binding to TNF receptor-associated factors TRAF1 and TRAF2)
*BTLA*	BTLA	B and T lymphocyte associated (receptor that relays inhibitory signals to suppress the immune response)
*CASP3*	Caspase 3	Executioner caspase/autophagy
*CASP6*	Caspase 6	Weak executioner caspase /cell cycle/B-cell proliferation
*CASP9*	Caspase 9	Initiator caspase (intrinsic pathway)/cleavage and activation of executioner caspases
*CD274*	PD-L1/CD274	PD1 ligand/ Inhibits T-cell activation and cytokine production
*CD28*	CD28	Delivers the critical costimulatory signal of T cells by binding to B7 (CD80/C86)/induces T-cell proliferation and cytokine production
*CD47*	CD47	Involved in the increase in intracellular calcium concentration that occurs upon cell adhesion to extracellular matrix
*CD58* (279 nt isoform)	CD58/LFA-3	Membrane protein/by binding its ligand CD2, its allows the adhesion and activation of both T lymphocytes and NK cells
*CDK2*	CDK2	Cyclin-dependent kinase 2/catalytic subunit of the cyclin-dependent protein kinase complex implicated in the G1 to S phase transition
*CDKN1B*	CDKN1B/p27KIP1	Cyclin dependent kinase inhibitor 1B
*CDKN1B*	CDKN1B/p27KIP1	Cyclin-dependent kinase inhibitor 1B
*CTLA4*	CTLA-4/CD152	Cytotoxic T-lymphocyte associated protein 4/competes with CD28 for binding to B7 (CD80/C86) thus reducing CD28-mediated T-cell costimulation
*DPP8*	DPP8	Dipeptidyl peptidase 8/pyroptosis
*DCK*	DCK	Deoxycytidine kinase/produces the monophosphate form of gemcitabine
*EBI2*	EBI2/GPR183	Epstein-Barr virus-induced gene 2/naive B cells, activated B cells (but not GC B cells), plasmablasts, plasma cells
*FOS*	FOS	AP-1 transcription factor
*FOXO3*	FOXO3	Forkhead box O3/triggers genes needed for cell death
*GATA3*	GATA3	GATA binding protein 3/transcription factor and important regulator of T-cell development
*GNA13*	GNA13	G protein subunit alpha 13 (confinement of B cell to the GC)
*ICOSLG*	ICOSL/CD275	ICOSL/involved in CD4 Tfh cell differentiation, T-cell-dependent B-cell responses and CD8 cytotoxic T-cell activation
*INPP5D*	SHIP1/INPP5D	Inositol polyphosphate-5-phosphatase D/negatively regulates the PI3K in B cells
*JAK2*	JAK2	Janus kinase 2 (central role in cytokine and growth factor signaling)
*MALT1*	MALT1	MALT1 paracaspase (caspase-like protease that plays a role in BCL10-induced activation of NF-kappaB)
*MAPK8*	JNK/JNK1	MAPK8/proliferation, differentiation, and transcription regulation/mediates immediate-early gene expression in response to cell stimuli
*MAPK9 (*4448 nt and 4598 nt isoforms*)*	MAPK9/JNK2	MAPK9/proliferation, differentiation, and transcription regulation/most closely related to MAPK8
*MAPK10*	JNK3	MAPK8/proliferation, differentiation, and transcription regulation/expressed in a subset of neurons in the nervous system
*MAPK14*	p38*α*	Proliferation, differentiation, and transcription regulation/activated by various environmental stresses and proinflammatory cytokines
*MKK6*	MKK6	Phosphorylation of p38*α*
*MLH1*	MLH1	mutL homolog 1/DNA mismatch repair
*MSH2*	MSH2	mutS homolog 2/DNA mismatch repair
*MSH6*	MSH6	mutS homolog 6/DNA mismatch repair
*MYD88*	MYD88	MYD88 innate immune signal transduction adaptor (essential signal transducer in the IL-1 and Toll-like receptor signaling pathway)
*NOTCH2*	NOTCH2	Notch signaling pathway
*PPMD1/WIP1*	WIP1	Protein phosphatase/inhibits p38*α* phosphorylation and activation, and through it the phosphorylation of p53, thus suppressing p53-mediated transcription and apoptosis
*PTEN*	PTEN	Phosphatase and tensin homolog (GCB-DLBCLs/BLs)/ dephosphoryation of the PI3K
*RAD51*	RAD51	RAD51 recombinase/homologous recombination and DNA repair
*SMAD1*	SMAD1	SMAD family member 1 (mediates the signals of the bone morphogenetic proteins, which are involved in a range of biological activities including cell growth, apoptosis, morphogenesis, development, and immune responses)
*SMAD2*	SMAD2	SMAD family member 2 (TGF*β* pathway)
*SMAD3*	SMAD3	SMAD family member 3 (TGF*β* pathway)
*SMARCA4*	BRG1/SMARCA4	SWI/SNF-related BAF chromatin remodeling complex subunit ATPase 4
*SOCS1*	SOCS1	SOCS1 (cytokine-inducible negative regulators of cytokine signaling)
*TERF1*	TERF1/TRF1	Telomeric repeat binding factor 1/inhibitor of telomerase
*TET2* (5279 nt isoform)	TET2	Tet methylcytosine dioxygenase 2/catalyzes the conversion of methylcytosine to 5-hydroxymethylcytosine
*TP53INP1*	TP53INP1	TP53INP1/involved in autophagic cell death
*TSC1*	TSC1	TSC complex subunit 1 (tumor suppressor, negative regulation of mTORC1)
*WEE1*	WEE1	G2 checkpoint kinase (inhibitory tyrosine phosphorylation of CDC2/cyclin B kinase)

DCK, deoxycytidine kinase.

## Conflict of interest

No author has an actual or perceived conflict of interest with the contents of this article.
